# Vehicle Maneuver Detection with Accelerometer-Based Classification

**DOI:** 10.3390/s16101618

**Published:** 2016-09-29

**Authors:** Javier Cervantes-Villanueva, Daniel Carrillo-Zapata, Fernando Terroso-Saenz, Mercedes Valdes-Vela, Antonio F. Skarmeta

**Affiliations:** Department of Communications and Information Engineering, Computer Science Faculty, University of Murcia, 30080 Murcia, Spain; javier.cervantes@um.es (J.C.-V.); daniel.carrillo2@um.es (D.C.-Z.); mdvaldes@um.es (M.V.-V.); skarmeta@um.es (A.F.S.)

**Keywords:** vehicle maneuver detection, accelerometer classification, mobile system

## Abstract

In the mobile computing era, smartphones have become instrumental tools to develop innovative mobile context-aware systems. In that sense, their usage in the vehicular domain eases the development of novel and personal transportation solutions. In this frame, the present work introduces an innovative mechanism to perceive the current kinematic state of a vehicle on the basis of the accelerometer data from a smartphone mounted in the vehicle. Unlike previous proposals, the introduced architecture targets the computational limitations of such devices to carry out the detection process following an incremental approach. For its realization, we have evaluated different classification algorithms to act as agents within the architecture. Finally, our approach has been tested with a real-world dataset collected by means of the ad hoc mobile application developed.

## 1. Introduction

Smartphones have been the center of most of the technological advances for the last decade. As a result, they are currently equipped with several embedded sensors like a GPS, accelerometer, gyroscope or magnetometer. This makes them suitable enablers to capture a wide range of contextual features, like weather [1] and traffic [2] conditions, or user behaviors [3]. Consequently, they are instrumental tools to develop mobile ubiquitous solutions [4,5].

During the last few years, a novel course of action intends to move such smartphone-based contextual perception to the vehicular domain [6,7,8]. More in detail, smartphones are used to enrich the sensing features of traditional situation-aware driving assistance systems (SADASs) [9]. This allows one to capture a wider range of driving events resulting in safer and more comfortable trips.

One common driving event to be perceived in a timely manner by SADASs is the current maneuver of a vehicle [10]. In that sense, some works already use the inertial and motion sensors of a smartphone, mounted in a car, to detect dangerous behaviors of the driver (e.g., speeding or drunk driving) [7,11] or rough road conditions [12]. However, little effort has been done so far to use such sensors to detect the vehicular maneuver in more varied scenarios.

Consequently, the present work focuses on detecting a vehicle’s maneuvers using the accelerometer sensor of a smartphone inside the vehicle on the basis of a taxonomy of four states, stopped, driving, parking and parked. Unlike previous solutions, these maneuvers have been defined from the point of view of a vehicle’s movement in an urban domain. Hence, they can be useful in different scenarios. Besides, the proposed system has been designed to locally run on a smartphone. Since this type of device has constrains in terms of battery, memory and processing capabilities, we deal with these limitations in two ways,

- The maneuver detection from accelerometer data is done by two interconnected classification agents following an incremental approach. This intends to reduce the general overload of the solution. In order to instantiate these agents, three classification algorithms have been studied, random forests (RF) [13], support vector machines (SVM) [14] and fuzzy rule-based classifiers (FRC) [15].
- In order to minimize the execution of the aforementioned agents, we have devised a lightweight mechanism that detects meaningful variations of the vehicle’s speed. Thus, only when a remarkable speed change is detected, the maneuver detection is launched. As we will see, this reduces even more the computational load of the proposal.

The present solution can be of great help in several domains. First of all, existing solutions for participatory vacant parking space management strongly depend on manual reports of users each time they occupy or leave a parking space (Wazypark, http://www.wazypark.com; Waze, https://www.waze.com). Since the proposed system detects the instant at which a vehicle is being parked, this automatic detection would be a useful feature to come up with more reliable solutions reporting available parking spaces in a city.

Secondly, the capability of the system to detect moving and stopped episodes of a vehicle can be useful for distributed traffic information systems in order to control the traffic state of a region of interest.

On the whole, the salient contributions of the present work can be summarized as follows: (1) a novel maneuver detection for vehicles using a smartphone’s acceleration measurements; (2) a mechanism to reduce the global overload of such detection; (3) a study of different algorithms for the accelerometer-based classification of vehicular maneuvers.

The remainder of the paper is structured as follows: an overview of the state of the art of vehicle-maneuver detection and accelerometer-based classification is put forward in Section 2. A detailed explanation of the proposed system is stated in Section 3. Then, Section 4 discusses the suitability of the candidate algorithms to implement the inner classifiers of the system. Next, Section 5 shows the final evaluation of the system. Finally, Section 6 puts forward the main conclusions of the work.

## 2. Related Work

In this section, we provide an overview about the two main domains related to this work, the usage of motion sensors for activity recognition and the detection of maneuvers in the vehicular context.

### 2.1. Activity Recognition Based on Accelerometer

The accelerometer sensor has been widely used to perceive the current activity of a person [16]. Depending on the device under consideration, we can distinguish among works that make use of wearable devices [17] and solutions that rely on smartphones [18].

In both cases, the general approach consists of extracting certain features of the measurements from the accelerometer and then applying different types of learning models to generate a final classifier able to infer the current behavior from a set of pre-defined ones. In that sense, the most common approach is to train the classifiers in a desktop machine before installing them in a smartphone [19]. However, alternative solutions propose to train such classifiers on the mobile phone in real time [18].

In this scope, a prominent line of research focuses on detecting the locomotion activity of a person (e.g., standing, walking, lying, climbing, jogging, and so on) [16]. In that sense, a palette of supervised learning models has been applied for that goal, like support vector machines [19], decision trees [20], K-nearest neighbors (KNN) [21] and naive Bayes [22]. Other works have also successfully applied statistical modeling methods. This is the case of hidden Markov models [23] or conditional random fields [24].

Our work is enclosed in a recent course of action that proposes to use the aforementioned learning methods within the vehicular or transportation domain using the accelerometer data of vehicle-mounted devices. For example, some works are already able to distinguish between motorized and non-motorized means of transport used by a person by processing the readings from his or her smartphone’s accelerometer with an acceptable confidence level [25,26].

Regarding these works, the present proposal centers on a different problem in the vehicular domain, as it does not distinguish among means of transport. On the contrary, provided that the user is using a motorized vehicle, it focuses on detecting the current kinematic state of such a vehicle. Thus, the information extracted by our proposal can complement the one from such mechanisms. For example, given that it is detected that a person is using a motorized means of transport by any of the aforementioned techniques, the present work can enrich such information with the current maneuver of the vehicle at each moment.

Finally, a plethora of solutions for driver profiling has also been proposed following a similar approach [7,11,27,28]. The key goal of these works is to use such information to detect particular risky situations during a trip, like turning-acceleration episodes [7,28], aggressive-normal [27] or drunk [11] driving.

Like these proposals, our work also intends to detect the activity that the driver is performing in each moment by means of a supervised learning method. However, the target activities are related to the current maneuver of the vehicle from a kinematic point of view. Consequently, unlike previous solutions, the present work is designed as a cross-domain vehicular maneuver detection that can be used beyond security purposes.

Furthermore, the present work also describes an incremental approach to perform maneuver detection that intends to minimize the computational load of the whole solution by reducing the global execution of the classifiers. This is an important advantage with respect to existing approaches, which assume the continuous execution of the classification models, bearing in mind the limitations in terms of the computation of current mobile platforms.

### 2.2. Vehicular Maneuver Detection

The detection of the current maneuver of the vehicle has been widely studied in the vehicular field as an important type of information used by collision-avoidance support systems. In that sense, the current or future kinematic state of a vehicle is instrumental so as to assess its associated risk in a scene. In this context, there already exists several solutions based on sets of different models capable of recognizing pre-defined vehicular kinematic states relying on different sensors and units installed in a vehicle (also known as Ego Vehicle, EgoV).

For example, [29] applies an unscented Kalman filter for curvilinear motions in an interactive multiple model (IMM) algorithm to keep track of a maneuvering vehicle. Similarly, [30] introduces a lateral and longitudinal maneuver predictor based on an IMM algorithm taking as input GPS/IMU measurements and a digital custom map. Moreover, in [31], the authors put forward a neuro-fuzzy architecture for maneuver prediction using a navigation unit composed of GPS, odometry and a gyro. Finally, [10] states a fuzzy rule-based classifier to detect the longitudinal maneuver of the EgoV by also using an on-board inertial measurement unit.

Even though our solution pursues a quite similar goal, it only depends on the acceleration measurements of a smartphone mounted in the EgoV. Since it does not depend on any onboard sensor or unit of the EgoV it can be regarded as a low-cost solution with respect to existing vehicle maneuver detection approaches.

## 3. System Design

This section is devoted to describing in detail the design of the proposed mechanism of vehicular maneuver detection based on the accelerometer. To do so, we firstly put forward the target maneuvers of the EgoV that the system is ready to detect.

### 3.1. Target Maneuvers

The present proposal assumes that the driving loop of a vehicle usually follows a simple pattern, the vehicle always starts in a stationary state, followed by a constant movement, which is only interrupted by stop episodes of different time lengths. Finally, such movement finishes in a parking state. This closes the loop, and the vehicle comes back to its initial stationary state.

Bearing in mind the aforementioned driving loop, the present work focuses on detecting the kinematic state of a vehicle by distinguishing among four possible maneuvers: parking (*PRK*), parked (*PRD*), stopped (*ST*) and driving (*DR*) , Ω={PRK,PRD,ST,DR}

**Figure 1:**
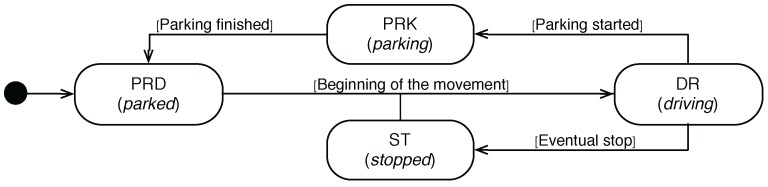
Target maneuvers and conceptual transitions among them.

As Figure 1 depicts, PRK represents the time period between the moment at which a vehicle remarkably decreases its speed to start the parking maneuvers and the instant when it eventually stops. PRD is the stationary state of its parking place, whereas ST represents the rest of stationary states of the vehicle due to, for example, red traffic lights. Finally, DR stands for the moving episodes of the vehicle.

### 3.2. System Architecture

**Figure 2:**
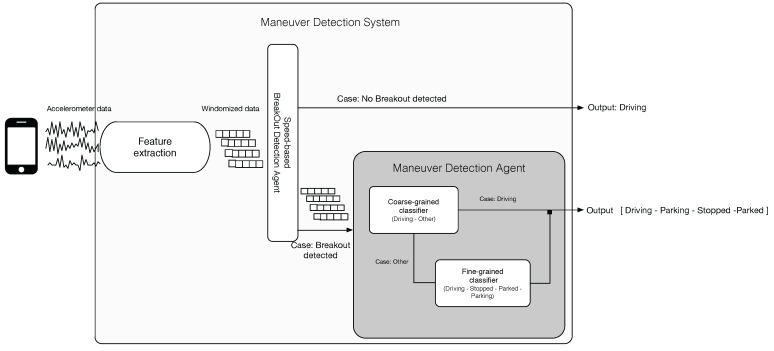
Architecture of the system.

In this section, we describe the architecture of the proposed system aiming at detecting the four maneuvers described above. This system has been designed to locally run in a smartphone, and as Figure 2 shows, it takes as input the data from the built-in accelerometer of the device and reports the current kinematic state of the vehicle where the smartphone is mounted.

In that sense, there are other sensors widely available in regular smartphones like GPS that could be also suitable for the classification goal of the system. However, the key benefit of the accelerometer sensor with respect to such sources is that there is much less battery draining when it is intensively used [32]. This is a key feature so as to come up with user-friendly applications. Furthermore, as has been described in Section 2, it has been successfully used for many classification domains.

As far as the inner structure of the system is concerned, it comprises a fine-grained and a coarse-grained classification agent. These two agents and the speed-based breakout detector agent (SBDA) work in a hierarchical procedure so as to avoid a large consumption of the computational resources of the host smartphone. For the sake of clarity, Algorithm 1 shows such a procedure. We describe in detail of each of its parts in the following sections.
**Algorithm 1:** Global procedure of the system.
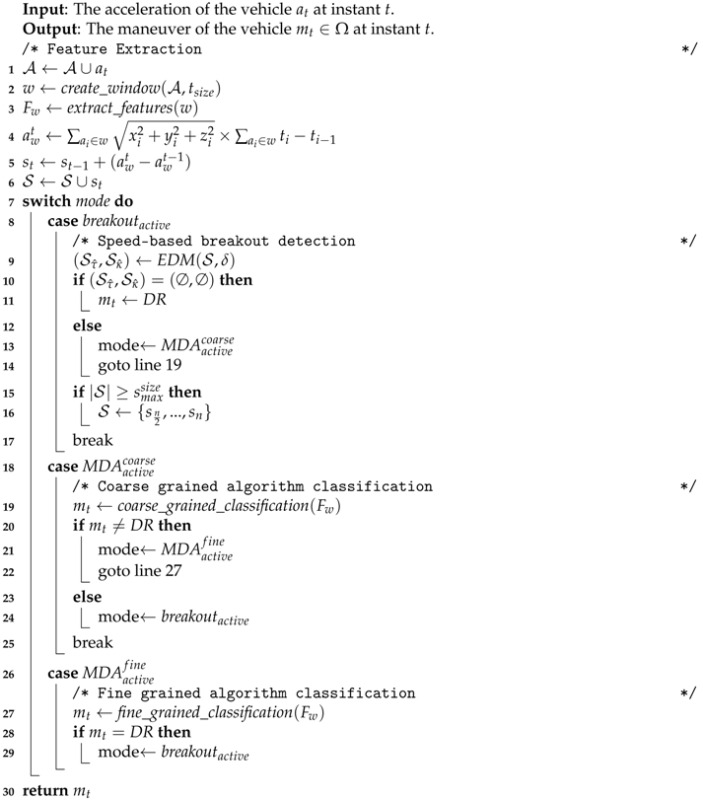



#### 3.2.1. Feature Extraction Module

The vehicle maneuver detection relies on the endless acquisition of the accelerometer data stream from the host smartphone, $A=\{a_{1},a_{2},..,a_{n}\}$, where $a_{i}=\left\{x_{i},y_{i},z_{i},t_{i}\right\}$ is a tuple with acceleration ($x_{i},y_{i},z_{i}$) at instant $t_{i}$. Then, A is continuously partitioned in time-based sliding windows *w* of length $t_{size}$ (Line 2 of Algorithm 1).

Next, for each window *w*, the feature extraction module (FEM) (see Figure 2) composes a set of time and statistical features $F_{w}$ (Line 3). To do so, the present work takes as a reference the analysis carried out in [19,25] about human and transport activity recognition. However, unlike these works, our approach is not driven to distinguish different transport-based activities, such as take the train, the car or walking. Nevertheless, their strategy can be also applied in our setting, taking into account that each target maneuver has a time length and can be treated as a distinct activity.

Furthermore, the FEM also uses each new *w* to approximate the vehicle’s speed at instant *j* ($s_{j}$) by means of the Euler method (Lines 4–5 of Algorithm 1). Next, each new $F_{w}$ feeds three parts of the system, the SBDA, the coarse-grained and the fine-grained classification agents (see Figure 2).

#### 3.2.2. Speed-Based Breakout Detection Agent

This agent analyzes the speed measurements, *s*, calculated by the previous module to detect abrupt changes in the EgoV’s speed that may represent potential shifts in its kinematic state. For example, a vehicle’s transition from *DR* to *ST* will be reflected in its speed. For that goal, this agent makes use of the E-divisive with median (EDM) algorithm [33]. This non-parametric algorithm allows timely detection of the changes in the speed mean using statistically robust metrics.

In a nutshell, and in our setting, the EDM algorithm takes as input the sequence of speed values incrementally generated by the FEM, $S=\{s_{1},s_{2},\ldots ,s_{n}\}$. Then, EDM splits such a sequence into two sub-subsequences $S_{\tau }=\left\{s_{1},s_{2},\ldots ,s_{\tau }\right\}$ and $S_{\kappa }=\left\{s_{\tau +1},s_{\tau +2},\ldots ,s_{\kappa }\right\}$, where 1<δ≤τ and τ+δ≤κ≤n.

Consequently, the two resulting sub-sequences $S_{\tau }$ and $S_{\kappa }$ comprise at least *δ* observations, and *τ* is the breakout point of both sub-sequences. The calculation of the best breakout point $\hat{\tau }$ can by done by solving a maximization problem,

$$\left(\hat{\tau },\hat{\kappa }\right)=\arg\max_{\tau ,\kappa }\tilde{Q}\left(A_{\tau },B_{\tau }\left(\kappa \right);\alpha ,\delta \right)$$

where $\tilde{Q}$ calculates the energy distance between two sets and *α* is a scale factor. This way, EDM not only obtains an estimate $\hat{\tau }$, but also its associated test statistic value $\hat{q}$. Given this and a predetermined significance level, the algorithm performs a permutation test to determine whether the reported breakout is statistically significant.

All in all, the speed-based breakout detection agent (SBDA) endlessly feeds the EDM algorithm with the current speed sequence *S* (Line 9 of Algorithm 1). If this algorithm does not report any significant breakout, the SBDA will assume that there is not any remarkable change in the EgoV’s speed and, thus, its kinematic state. Consequently, it automatically generates the system’s output as *DR* (Lines 10–11). This assumption is based on the intuitive idea that *DR* is the most common state in Ω when a vehicle is being driven.

In case a relevant speed breakout is detected, then it might be a sign that the EgoV’s maneuver has actually changed or is about to change. Therefore, in order to clearly perceive the (potentially) new maneuver of the vehicle, the SBDA activates the classification agents in order to compose the system’s outcome (Lines 13–14). At this point, all of the features sets $F_{w}$ are directly delivered to the maneuver detection agent (MDA).

Finally, the SBDA also controls the whole size of S (Lines 15–16 of Algorithm 1). If such a size exceeds a particular threshold, then the set is restricted to its $\frac{n}{2}$ last values.

#### 3.2.3. Maneuver Detection Agent

The core of the proposed system is the MDA that comprises two classification models. As Figure 2 depicts, both models are hierarchically organized in order to reduce the number of times they are executed.

The top level of the hierarchy is composed of a coarse-grained classifier (see Figure 2). This model runs when the SBDA reports a significant speed change (Lines 18–25 of Algorithm 1). The goal of this first model is to detect whether the vehicle still remains in the *DR* state or, on the contrary, has actually moved to a different non-driving maneuver (*ST*, *PKR*, *PKD*). For that goal, it classifies the incoming $F_{w}$ tuples into *DR* or non-*DR*. In that sense, it only considers a sub-set of $F_{w}$. By reducing the number of input features, we intend to reduce the complexity and computational load of this first classifier.

Provided that the outcome of this first classifier is a non-driving maneuver (Lines 20–24), the second-level classifier will be activated (see Figure 2). This model is in charge of uncovering the actual maneuver of the EgoV in Ω. To do so, a classification of the incoming $F_{w}$ tuples is carried out (Lines 26–29). Due to the fact that in this case, a high level of accuracy is required, this classifier takes as input all of the features in each $F_{w}$.

Lastly, if the maneuver returned by the coarse-grained or the fine-grained classifier is DR, the SBDA is re-launched and the MDA stopped until a new abrupt change is detected (Lines 24 and 29).

### 3.3. System Orchestration

**Figure 3:**
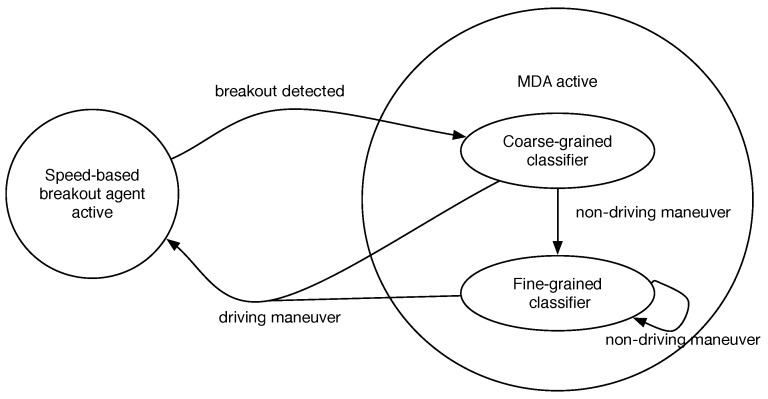
Orchestration of agents and models.

For the sake of completeness, the orchestration of the SBDA and the MDA is shown in Figure 3. As we can see, the key goal of such a cooperation is to reduce as much as possible the number of times the MDA is launched. This is because the continuous execution of its classifiers in a mobile platform might be computationally draining.

In that sense, the fine-grained classifier is the most complex element of the system in terms of computation, as it makes use of all of the calculated features and should be able to distinguish among all maneuvers in Ω. Hence, it is only executed when both the SBDA and the coarse-grained classifier infer that a meaningful change in the kinematic state of the vehicle has likely occurred.

## 4. Classifiers Generation

Once the design of the system has been devised, the next step is to study which particular models should play the role of fine- and coarse-grained classifiers within the MDA. For that goal, we evaluated and compared different classification algorithms. The results of this study are presented in this section.

### 4.1. Target Classification Algorithms

We have considered three different data-driven and supervised classification algorithms, namely random forest (RF), support vector machines (SVM) and fuzzy rule-based classifier (FRC). While the first two algorithms are well-known solutions to uncover human activities with accelerometer data, FRC has not been fully explored in this field. An overview of each method is put forward next.

#### 4.1.1. Random Forest

This classifier consists of a combination of decision trees as described in [13]. It takes advantage of two powerful machine-learning techniques: bagging [34] and random feature selection [35,36].

The bagging method allows reducing the variance of the model without increasing its bias. This makes the predictor less sensitive to noise and less prone to overfitting. Besides, the random feature selection is the key difference between RF and regular bagging predictors. It involves randomly selecting a small group of input features at each tree’s node. Therefore, unlike other similar approaches, such as adaptive boosting (AdaBoost), RF is more robust to outliers and noise, which is very convenient when dealing with accelerometer-based data [13,25].

#### 4.1.2. Support Vector Machine

Support vector machine is a classifier based on statistical theory that works by defining hyperplanes to separate data into different classes [14]. Each defined hyperplane divides the data into two classes and tries to leave the maximum margin from both. In case that training data are not linearly separable, then it is common to combine it with kernel functions that transform the original data to a space of higher dimensions in order to find optimal hyperplanes there more easily.

#### 4.1.3. Fuzzy Rule-Based Classifier

This kind of model can be extracted from data following the approach described in [15]. This process is called fuzzy modeling, and it has been widely applied for regression problems. Since the goal in our setting is to perform a classification task, it can be approached as a regression problem with only a set of possible output values if the set of predefined maneuvers is converted into a set of numbers. Having this idea in mind, a set of fuzzy rules with fuzzy sets in the antecedents and first order polynomials of the input features as consequents can be used for our maneuver classification problem.

The fuzzy modeling process is composed of two main steps. The goal of the first step is the identification of the rule antecedents. For this aim, a fuzzy clustering technique is applied to the input-output dataset. Then, the detected fuzzy clusters are projected into each one of the input feature axes; afterwards, these projections are approximated by means of Gaussian bells, the obtained bells being the fuzzy sets composing the rule antecedents.

The second step is the identification of the rule consequents; that is, the identification of the coefficients of the polynomials composing the rule consequents. In the current approach, this is done by the application of the least square estimator (LSE).

### 4.2. Data Collection

**Figure 4:**
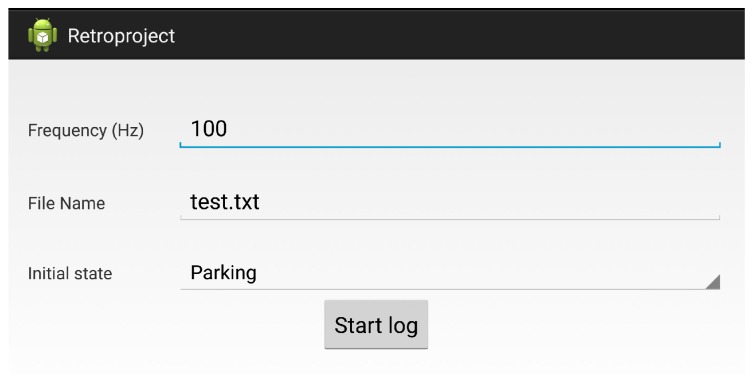
Screenshots of the android app used for data collection.

In order to collect a reliable dataset to train the three algorithms described above, we developed an Android application to read, store and label with its corresponding maneuver the measurements from the accelerometer. The application’s screen-shoot is shown in Figure 4.

**Figure 5:**
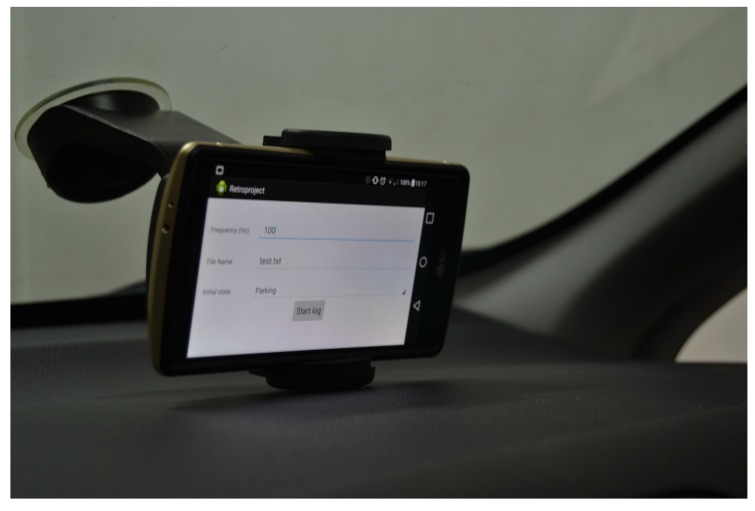
Smartphone installation for the data collection campaign.

**Figure 6:**
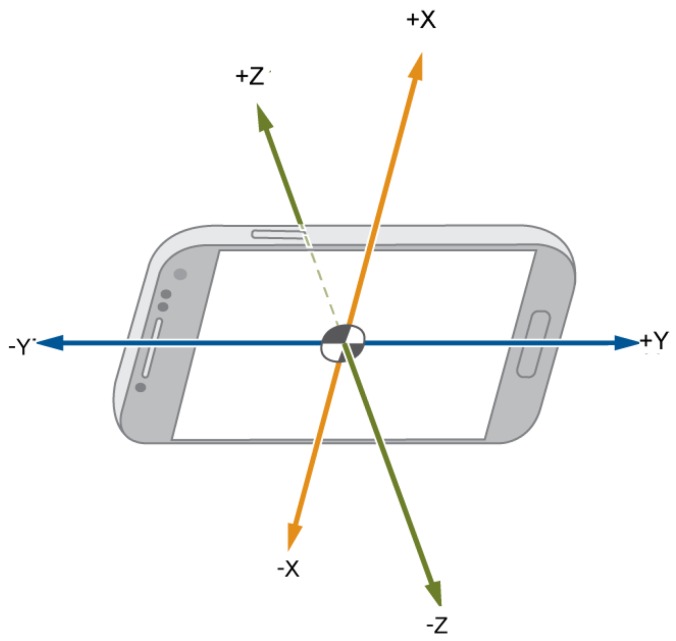
Accelerometer axes’ orientation during the data collection campaign.

We then mounted a smartphone running the aforementioned application in a vehicle. We covered with such a vehicle seven different urban circuits to generate a palette of driving situations. In all of the circuits, two person were involved. While the driver, who was always the same person, drove the vehicle, the other person used the application to label the data by using the aforementioned dataset. During the collection campaign, we used an LG G4 smartphone equipped with an accelerometer sensor with a resolution of 0.00119 m/s^2^ and a 100-Hz frequency. Figure 5 shows how the smartphone was mounted for this campaign, whereas Figure 6 depicts the resulting orientation of accelerometer’s axes of such an installation.

As a result, the seven circuits comprised a dataset with 110,200 timestamped and labeled accelerometer readings. In addition to that, we removed the gravity from these measurements to get realistic values of the vehicle movement. This is because when we keep the gravity acceleration, each acceleration component is affected with an increment that does not correspond with its real relative value. Finally, the distribution of the circuits with respect to the maneuver labels is shown in Table 1. For the sake of clarity, the name of these circuits just corresponds to the order in which they were covered; they do not represent any physical meaning.

### 4.3. Classifiers Training

Once we collected the whole dataset, the next step was to train each of the three algorithms in order to compose a set of classification candidates to act as coarse-grained and fine-grained classifiers.

#### 4.3.1. General Setup

Here, we describe the configuration details of the training of the algorithms.

##### Windows Generation

In order to generate the windows *w* from the collected data, we applied a time-based sliding window with $t_{size}$ of 0.5 seconds and 50% overlapping. Due to the fact that the accelerometer sensor was sampled to 100 Hz, each window contained over 50 samples. In addition to that, we also controlled that each window contained only readings labeled with the same maneuver to ease the training of the classifiers.

##### Feature Extraction

For each window *w*, Table 2 shows its set of 13 computed features $F_{w}$, where (x,y,z) refers to the particular accelerometer axes. These features have been repetitively used in the literature [18]. In that sense, $a_{total}$ is the acceleration obtained with the square root of the squared components of acceleration, and *s* is the vehicle speed calculated as has been put forward in Section 3.2.1. As we will see later, each of the two classifiers of the system is fed with a different subset of $F_{w}$.

##### Maneuver Fusion

In the original maneuver set Ω, *PRD* and *ST* actually represent the same kinematic state of the EgoV. Hence, for the present comparison, both maneuvers have been merged into a single stationary one (*STA* ). Thus, the resulting maneuver set turned into $\Omega^{\prime }=\left\{PRK,STA,DR\right\}$.

Given such a fusion, discovering the particular stationary maneuver (*PRD* or *ST*) if the system generates *STA* as the current state is possible by considering the previous maneuver detected by the system. This way, before *PRD*, the EgoV should be in *PRK*, whereas before *ST*, the EgoV should remain in the *DR* state. Consequently, the sequences {PRK→STA} and {DR→STA} will be automatically translated into {PRK→PRD} and {DR→ST} before being delivered by the system as the output.

#### 4.3.2. Classifiers Configuration

The default configuration of each algorithm for the comparison is shown in Table 3.

Regarding FRC, it was also necessary to perform a numerization of the output label of every tuple. In this case, we applied a one to one numerization, as it does not incorporate additional complexity in the problem. However, this technique can only be applied whether some kind of total order can be established among the nominal values. In our case, it is obvious that we can define the partial order, 1-STA, 2-PRK, 3-DR, given that a stopped vehicle (1) needs to un-park (2) so as to reach a cruise velocity (3).

#### 4.3.3. Training Method

The training of the three proposed classifiers has been done with a varied data distribution. On the basis of the seven collected circuits, we defined three experiments splitting the data into training and evaluation sets. Table 4 shows the composition in circuits of each experiments.

For E1, $circuit_{5}$ was selected for evaluation, because it contains the higher number of samples and multiple state changes (see Table 1). Regarding E2, we used $circuit_{4}$ as the evaluation circuit due to the fact that it has the largest amount of *PRK* and *PRD* samples. Finally, $circuit_{3}$ was used in E3 to prove the quality of the system classifying a great number of *PRK* samples.

Lastly, the three algorithms have been trained by following a repeated *k*-fold cross-validation using the six training circuits of each experiment with the *k* parameter set to 10 and the number of repetitions to five. Consequently, in each iteration, 90% of the training circuits’ tuples were used to train and the remaining 10% to test. We opted for this approach instead of a static division of the dataset into training and testing due to the large number of *DR* maneuvers with respect the other ones. Such an unbalanced distribution of labels might lead to biased models if we used the aforementioned static division.

### 4.4. Classifiers Results

In this section, we provide the results obtained by the three algorithms under consideration. In order to discard a potential over-fitting of the models, we show both the training and the evaluation errors of the candidates.

#### 4.4.1. Coarse-Grained Classifier

Recall that this first model provides a binary classification by detecting whether the vehicle is in DR or any other state, and it should only consider a few features of $F_{w}$ as input to reduce its computational load. Bearing this goal in mind, we performed a filter-based feature selection to generate a sub-set of $F_{w}$ to feed the first model candidates. It is important to note that, for this procedure, the non-driving maneuvers (*PRD*, *ST* and *PRK*) were fused into a single artificial NoDR one giving rise to the maneuver set $\Omega^{\prime \prime }=\left\{DR,\mathrm{NoDR}\right\}$.

As a result of this selection process, four features were selected as the input of the three algorithms under consideration, s,VAR(x),VAR(z), and the accumulative mean of VAR(y). In that sense, we observed that the variance of the acceleration components provides useful information about the kinematic state of the EgoV. In particular, a high variance corresponds with moments where the EgoV’s speed covers a wide range of values that usually corresponds to a *DR* state. Apart from that, a low variance represents episodes when the EgoV is in a low speed range, like the *PRK* or *STA* states.

Regarding the accumulative mean of VAR(y), this feature has been selected because it smooths the variance of *y*. This is because VAR(y) tends to zero and remains at such a value when the EgoV is stopped. Such a decrease of the variance occurs in a smooth manner. However, for the prompt detection of a stationary state, it is more useful that the convergence of VAR(y) towards zero occurs more quickly. Since the accumulative mean of such a feature is less noisy because it retains the average value of the data and discards random peaks, its convergence towards zero is faster than VAR(y), and thus, it becomes a more useful feature for the classifiers.

**Figure 7:**
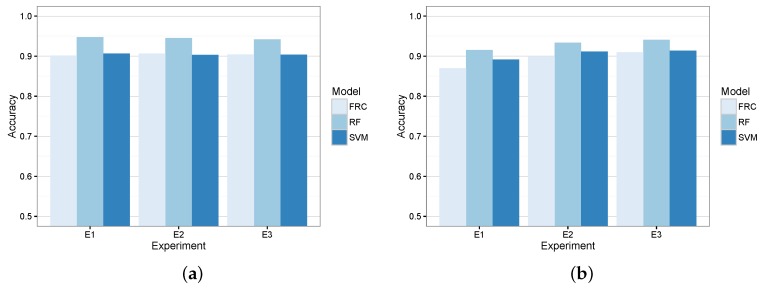
Coarse-grained classifier candidates’ accuracy. (**a**) Training error; (**b**) evaluation error.

Figure 7 shows the resulting models’ accuracy in terms of training and evaluation errors when acting as a coarse-grained classifier. According to these results, we did not observe signs of overfitting, and RF obtained lower errors in the three experiments by correctly classifying more than 90% of the tuples. Furthermore, Table 5 shows the confusion matrices of the three classifiers for the evaluation circuits of the experiments.

Results from such a table show that RF overcomes the other two candidates. In particular, this algorithm was able to correctly classify between 94% and 93% of the coarse-grained maneuvers. On the contrary, the other two candidates achieved a significantly lower accuracy.

For the sake of completeness, Table 6 shows the average sensibility and specificity of the three models. In this case, the DR maneuver is regarded as the positive state and *NoDR* as the negative state. It also depicts the accuracy, ACC ($=\frac{TP+TN}{TP+TN+FP+FN}$), sensitivity, SEN ($=\frac{TP}{TP+FN}$), and specificity, SPE ($=\frac{TN}{TN+FP}$), of the models. As we can see, RF achieved the best ACC and SEN rates and an acceptable SPE value. This is consistent with the results shown in previous confusion matrices.

#### 4.4.2. Fine-Grained Classifier

This second-level classifier is in charge of detecting the specific maneuver of the vehicle in the case that the SBDA reports that a meaningful change of the vehicle’s speed has just occurred, and the coarse-grained classifier reports that the current maneuver of the vehicle is different from *DR*. Consequently, this fine-grained classifier plays a crucial role for the whole accuracy of the system.

**Figure 8:**
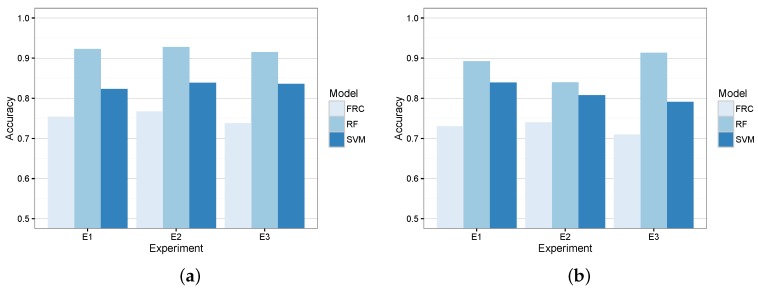
Fine-grained classifier candidates’ accuracy. (**a**) Training error; (**b**) evaluation error.

For that reason, the three candidates have been trained with all of the features described in Table 2. Figure 8 depicts the training and evaluation error of the three candidate algorithms in each of the devised experiments. As we can see, there was no overfitting, and RF obtained the most promising results for this second model. In fact, RF generated the models that kept a similar accuracy for both the coarse-grained and fine-grained classifiers.

For the sake of completeness, Table 7 shows the confusion matrices of the three candidates as fine-grained classifiers for the evaluation circuit of each experiment. Despite the fact that FRC achieved the best accuracy for the *PRK* maneuver, the RF model obtained a better general accuracy for the three experiments and maneuvers.

Lastly, Table 8 depicts the average number of TP, TN, FP and FN of the candidates in the three experiments with respect to each maneuver in $\Omega^{\prime }$. It also depicts the SEN, SPE and ACC of the models. In this case, results also show that RF achieved the best ACC in two out of three maneuvers and quite high and stable SEN and SPE values for the three maneuvers.

### 4.5. Classifiers Comparative

Since the evaluation results stated above cover different classifiers and multiple experiments, we cannot just compare the average accuracies of the models. Such measurements might contain outliers and depend on the data.

As a result, we formally compare the three classifiers with the analysis of variance (ANOVA) and the Dunn test. By means of these tests, we computed the *p*-value of the null hypothesis that the evaluation results of the classifiers are different.

While ANOVA was used to detect whether meaningful differences actually exist between the three classifiers, the Dunn test was applied to uncover the particular differences among pairs of classifiers. Furthermore, we used the Levene and the Shapiro–Wilk tests to confirm the homoscedasticity of the evaluation results and the fact that they follow a normal distribution. These are two requirements for a dataset to be evaluated with the ANOVA test.

#### 4.5.1. Coarse-Grained Classifier

Regarding the comparison of the three algorithms when they acted as coarse-grained classifiers, Table 9 shows the *p*-values of the Levene and Shapiro–Wilk tests that confirmed the homoscedasticity and the normal distribution of the evaluation results.

Next, we launched the ANOVA test over the evaluation results reporting 0.00317 as the *p*-value. This indicates that meaningful differences exist among the results of the classifiers. Hence, we finally made use of the Dunn test discovering the particular differences between the classifiers whose results are contained in Table 10.

According to the results of this table, the only two classifiers with a different behavior are RF and FRC because their associated *p*-value (0.03) rejects the null hypothesis. Moreover, the negative difference of their evaluation means indicates that RF provides better classification capabilities than FRC as a coarse-grained classifier. Lastly, the test does not indicate a significant difference between either SVM and RF or SVM and FRC.

##### Selected Classifier

Keeping in mind the results of the comparison described above, we firstly discarded the FRC model as the coarse-grained classifier. Consequently, we had to decide between SVM and RF as the final classifier for the first level of the MDA. Since the Dunn test indicates certain similarities between both models, we finally opted for RF because its evaluation results were slightly better than SVM, as was described in Section 4.4.

#### 4.5.2. Fine-Grained Classifier

For the second classifier of the MDA, we followed the same procedure to compare the models. Thus, Table 11 shows the Levene and Shapiro–Wilk tests of the evaluation results of the models acting as a fine-grained classifier. They confirm the normal distribution and homoscedasticity of the results.

Since the ANOVA test returned a *p*-value of 0.00346, we eventually executed the Dunn test, whose results are shown in Table 12.

Again, RF and FRC exhibited a different behavior with a *p*-value of 0.03. Besides, the test also confirmed the best classification accuracy of RF with respect to FRC and the lack of significant differences between either SVM and RF or SVM and FRC.

##### Selected Classifier

To sum up, RF was chosen as the fine-grained classifier instead of SVM by considering the evaluation results described in Section 4.4.

## 5. System Evaluation

Once we selected the concrete models to implement the two layers of the MDA, we eventually evaluated the resulting system. To do so, we used the evaluation circuits of the three experiments already described in Section 4.3.3.

One of the key innovations of the present proposal is the usage of a mechanism to monitor meaningful changes of the vehicle’s speed to launch or not the MDA. Thus, we have evaluated the actual suitability of such a mechanism.

For that goal, we compared the proposed system with the SBDA enabled and a slight modification that does not include this agent. Such an alternative only comprises the fine-grained classifier within the MDA, which endlessly processes the incoming features $F_{w}$. Since this alternative requires the continuous execution of the fine-grained classifier, it implies a higher computational load.

**Figure 9:**
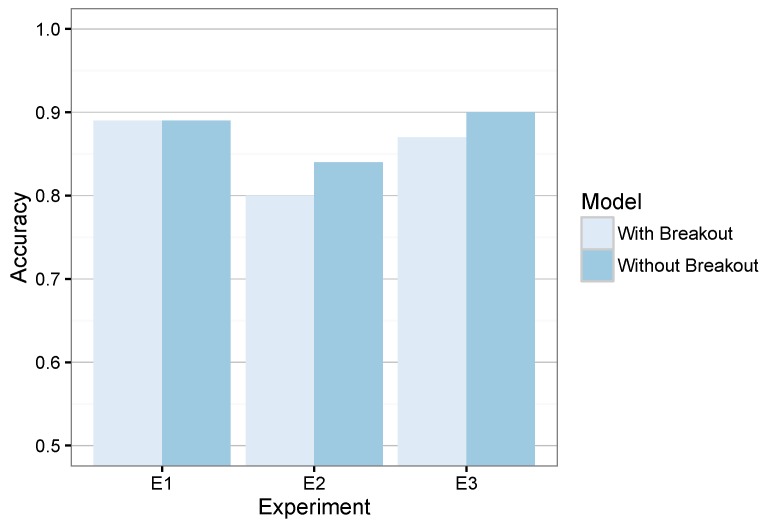
Evaluation error of the system when the speed-based breakout detection is activated or not.

When we compared our system and its alternative, we obtained the results shown in Figure 9. On average, the system accuracy with and without breakout is similar, while our approach also saves execution cycles.

Despite this reduction, Table 13 also shows that the speed change detection achieved its desired goal because it reduced the number of executions of the MDA by around 25%. Consequently, it is necessary to define a trade-off between accuracy and potential execution savings.

More in detail, Table 14 shows the confusion matrix of the system with and without the breakout mechanism. From this matrix, we observed that the most conflictive maneuver was *PRK*. In this case, the system suffered from the highest failure rate. This is because during a parking phase, the EgoV suffers similar changes of acceleration along the horizontal axis as in the driving phase. Besides, during the instant at which the EgoV is changing its movement forwards or backwards, it practically remains stationary for a moment, which can be misclassified as an *STA* maneuver.

Furthermore, the slight difference of accuracy when the breakout is disabled or enabled is suitable if we take into account the execution-cycle saving observed in Table 13 when the breakout is enabled.

**Figure 10:**
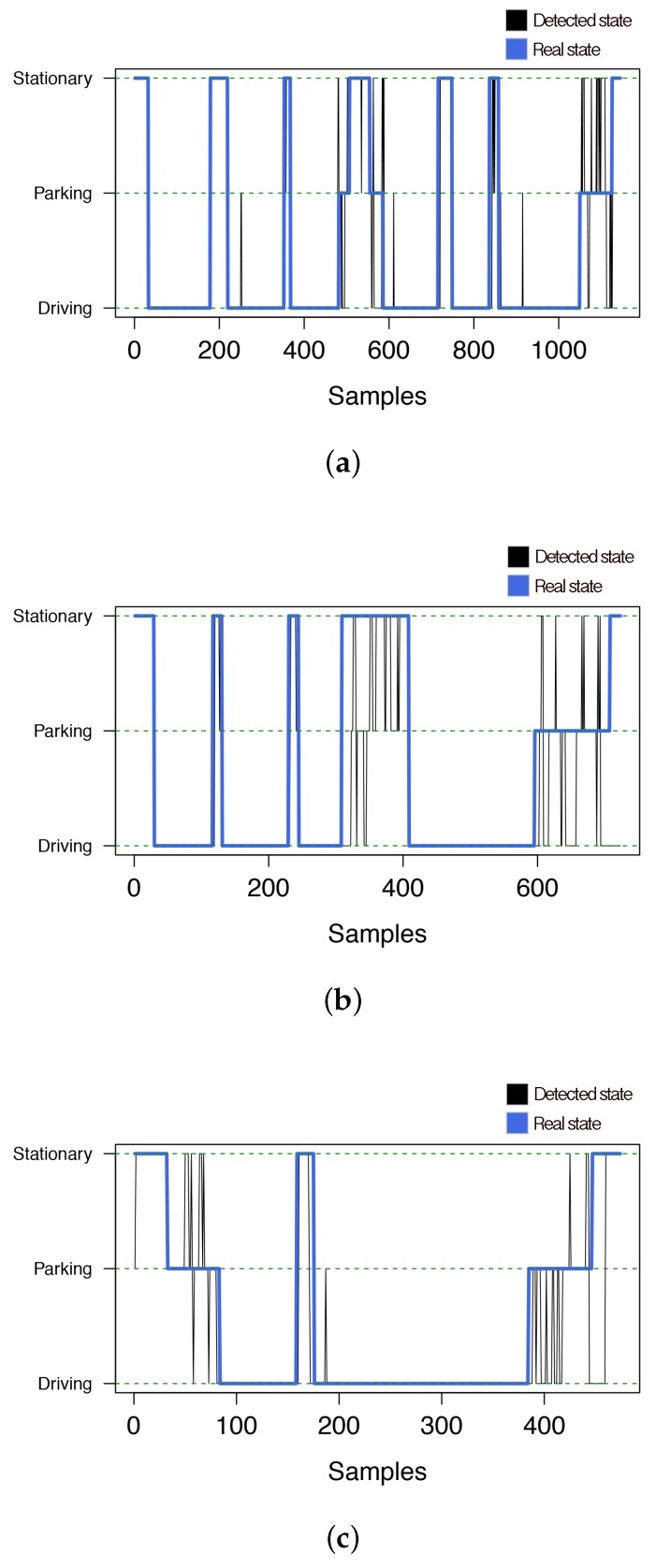
System predictions using breakout detection for the three target experiments. (**a**) Experiment E1; (**b**) Experiment E2; (**c**) Experiment E3.

Finally, Figure 10 shows the maneuver inferred by the system and the real one as time proceeds. As we can see, this figure confirms that most of the erroneous classifications were related to *ST* and *PRK* episodes, whereas it accurately perceived the rest of the *DR* episodes.

### 5.1. GPS Addition Evaluation

In order to evaluate the effect on the system’s accuracy of adding new sources of information, we decided to slightly modify the proposed system so that it was able to also read the GPS measurements of the smartphone. Thus, the current speed of the EgoV was not estimated by means of the accelerometer measurements, but directly extracted from the values returned by the GPS sensor. Then, such speed values were the ones that fed the SBDA and the two classifiers of the system.

For this evaluation, we generated two versions of the system, the one only using accelerometer data and the new one using both accelerometer and GPS data. In both cases, RF was the algorithm implementing the two classifiers of the system, since it was the one that provided the best accuracy according to the study in Section 4.

We also designed a new experiment comprising three new circuits for training and a new one for evaluation. In this case, we collected the readings coming from the accelerometer and GPS sensors of the smartphone whose model was the same as in the evaluation in Section 4 (LG G4). The four circuits comprised 197,887 instances (171,181 for training and 26,706 for test). The training procedure was also a k-fold cross-validation with the *k* parameter set to 10 and the number of repetitions to five.

Regarding the input features, they were the same as the ones in the evaluation of Section 4, but in this case, *s* was the speed directly returned by the GPS sensor.

Firstly, we individually evaluated the impact of the GPS-based speed in the two types of classifiers of the system. In that sense, Table 15 shows the confusion matrix of the coarse-grained classifier for the two versions of the system. We can see that including GPS data in the classification loop remarkably improves the capability of the system to detect non-driving states. This is because the EgoV’s speed estimation is more accurate when using GPS, and thus, this feature allows the classifier to better distinguish among states.

Regarding the fine-grained classifier (Table 16), we can see that the version using GPS remarkably improves the detection of the *STA* state with respect to the version using only accelerometer data. The rationale of this increment has also to do with the speed estimation. Since the detection of a stationary state is strongly related to EgoV’s low speed, the better estimation of this value makes the system more capable of accurately perceiving such a maneuver.

Finally, Table 17 depicts the general confusion matrix of the two versions of the whole system. Results confirm that the slight improvement of the GPS + accelerometer version affects the *DR* and *STA* states where the EgoV’s speed plays a key role (high speed for *DR* and low speed for *STA*).

Despite this improvement, we should consider the impact of using the GPS sensor on the smartphone’s battery because, as has been already mentioned, it is a rather battery-draining sensor. Consequently, a study of the trade-off between required accuracy and the associated energy consumption must be carried out in each particular deployment of the system.

### 5.2. Multi-User Usage Evaluation

Since the present system has been designed to mainly run on smartphones, it is intended to provide a personal detection of the maneuvers performed by the driver. In that sense, all of the training, testing and evaluation datasets used throughout all of the previous evaluations were related to a single and unique driver.

However, we have also briefly studied the performance of the system when it is used by a driver who is different from the one who generated the dataset that trained and composed the system. Thus, the goal of this evaluation is to give insight into the feasibility of coming up with a system that is suitable for a group of users instead of a single one. This multi-user solution might increase the potential exploitation of the system.

Consequently, we launched a new data-collection campaign by using the same mobile application to gather the datasets. Unlike the previous studies, this time, the person playing the driver role was different. As a result of this new campaign, a new dataset comprising 46,494 instances was generated. Table 18 depicts the details of such a dataset.

Next, we fed the system generated with the previous driver’s dataset, whose results were put forward at the beginning of Section 5, with this new dataset. This way, we evaluated the system with a dataset from a user different from the one that generated the system.

Table 19 shows the confusion matrix of the system when it was fed with this new dataset. Furthermore, for the sake of completeness, we also include the system’s matrix that we obtained when the system was trained and evaluated with the same driver (the average results in Table 14 for SBDA enabled).

As we can see from these results, the performance of the system remarkably drops when it is used by a different driver than the one for which the system was trained. This decrease is specially noticeable for *PRK* and *STA* for which only about 2% of the maneuvers were correctly classified.

This decrease is because during the training phase, the collected dataset is defined by certain personal features of the driver when it comes to using his or her vehicle like, for example his or her tendency to roughly park his or her car or to accelerate very fast when the vehicle is stopped. This type of underlying driving behavior of a person is indirectly learned by the system and, thus, makes the system less accurate when a different user makes use of it.

## 6. Conclusions

The sensor equipment of smartphones now allows one to capture more and more features of its surrounding environment, allowing one to develop a wide range of context-aware and pervasive solutions. In particular, the vehicular domain can benefit from such mobile platforms in many different ways to come up with innovative solutions to improve the traveling experience.

In this frame, the present work puts forward a novel mechanism to detect the current maneuver of a vehicle by processing the accelerometer readings of a smartphone. By means of a hierarchy of classifiers and the automatic detection of speed changes, the system is able to accurately perceive the vehicle’s kinematic state. Moreover, we have also considered the limitations of mobile platforms when it comes to coping with computationally-greedy applications.

For its realization, we have trained and compared three different supervised learning algorithms to study which one was the most suitable for the proposed architecture. The results proved that random forest was the best option to implement the two-level classifiers of the system. In addition to that, the final evaluation of the system confirmed that the detection of speed changes to activate the classifiers slightly reduces the accuracy of the whole system, but on the contrary, provides a lighter solution in terms of computational needs.

Finally, future work will focus on the integration of other common sensors of a smartphone, like the gyroscope, in order to improve the perception capabilities of the whole system.

## Figures and Tables

**Table 1:** Distribution of instances among maneuvers and circuits in terms of percentage and total number (in brackets).

Maneuver	*DR*	*PRD*	*PRK*	*ST*	Total
*circuit*_1_	68.38 (11,402)	14.51 (2419)	15.19 (2533)	1.93 (321)	16,675
*circuit*_2_	63.21 (5235)	18.97 (1571)	12.48 (1034)	5.34 (442)	8282
*circuit*_3_	59.85 (7141)	12.69 (1514)	23.84 (2844)	3.62 (432)	11,931
*circuit*_4_	60.39 (10,975)	20.10 (3653)	15.48 (2813)	4.04 (734)	18,175
*circuit*_5_	69.78 (20,112)	8.78 (2530)	11.61 (3345)	9.83 (2833)	28,820
*circuit*_6_	73.24 (8487)	8.99 (1042)	13.53 (1568)	4.24 (491)	11,588
*circuit*_7_	64.04 (9432)	11.89 (1751)	17.80 (2622)	6.27 (924)	14,729
Total	72,784	14,480	16,759	6177	110,200

**Table 2:** List of features $F_{w}$.

Domain	Features
Time Statistical	speed (s)
	mean ($\mu \left(x\right),\mu \left(y\right),\mu \left(z\right),\mu \left(a_{total}\right)$)
	variance (VAR(x),VAR(y),VAR(z))
	accumulative median ($VA\tilde{R}(y)$)
	standard deviation ($\text{STD}\left(x\right),\text{STD}\left(y\right),\text{STD}\left(z\right),\text{STD}\left(a_{\text{total}}\right)$)

**Table 3:** Algorithms default configuration. FRC, fuzzy rule-based classifier.

Algorithm	Parameter	Value	Meaning
RF	*n_trees_*	500	Max. number of trees to be generated
	*coeff*	*gini*	Contribution measurement
SVM	*model*	linear	Model for classification
	*C*	1	Violation threshold
FRC	*n_rules_*	[2:8]	Max. number of rules to be generated
	*m*	2	Cluster’s *fuzziness*

**Table 4:** Summary of experiments and circuits.

Experiment	Training	Evaluation
E1	$circuit_{1,2,3,4,6,7}$	$circuit_{5}$
E2	$circuit_{1,2,3,5,6,7}$	$circuit_{4}$
E3	$circuit_{1,2,4,5,6,7}$	$circuit_{3}$

**Table 5:** Confusion matrix of the candidates when acting as a coarse-grained classifier in terms of percentage and number (in brackets) of correctly-classified instances.

		*RF*		*SVM*		*FRC*	
Exp.	Man.	*DR*	*NoDR*	*DR*	*NoDR*	*DR*	*NoDR*
E1	DR	0.94 (750)	0.15 (45)	0.90 (724)	0.13 (46)	0.88 (704)	0.12 (40)
	*NoDR*	0.06 (52)	0.85 (298)	0.10 (78)	0.87 (297)	0.12 (98)	0.88 (303)
E2	DR	0.97 (425)	0.13 (36)	0.96 (418)	0.16(45)	0.92 (400)	0.09 (27)
	*NoDR*	0.03 (12)	0.87 (250)	0.04 (19)	0.84(241)	0.08 (37)	0.91 (259)
E3	DR	0.98 (278)	0.12 (22)	0.94 (267)	0.13 (24)	0.93 (264)	0.05 (10)
	*NoDR*	0.02 (6)	0.88 (168)	0.06 (17)	0.87 (166)	0.07(20)	0.95(180)

**Table 6:** Sensitivity (SEN), specificity (SPE) and accuracy (ACC) of the candidates for the coarse-grained classifier along with their number of true positives (TP), false positives (FP), true negatives (TN) and false negatives (FN).

	*RF*	*SVM*	*FRC*
TP (*DR* as *DR*)	1453	1409	1368
FN (*DR* as *NoDR*)	70	114	155
TN (*NoDR* as *NoDR*)	716	704	742
FP (*NoDR* as *DR*)	103	115	77
SEN	**0.95**	0.92	0.90
SPE	0.87	0.86	**0.91**
ACC	**0.92**	0.90	0.90

**Table 7:** Confusion matrix of the candidates when acting as a fine-grained classifier in terms of percentage and number (in brackets) of correctly-classified instances.

		*RF*			*SVM*			*FRC*		
Exp.	Man.	*DR*	*PRK*	*STA*	*DR*	*PRK*	*STA*	*DR*	*PRK*	*STA*
E1	*DR*	0.96 (774)	0.25 (33)	0.07 (15)	0.94 (753)	0.31 (41)	0.14 (30)	0.89 (713)	0.15 (20)	0.10 (21)
	*PRK*	0.03 (21)	0.49 (65)	0.06 (13)	0.04 (36)	0.24 (32)	0.02 (5)	0.11 (85)	0.54 (71)	0.18 (37)
	*STA*	0.01 (7)	0.26 (34)	0.87 (183)	0.02 (13)	0.45 (59)	0.83 (176)	0.00 (4)	0.31 (41)	0.73 (153)
E2	*DR*	0.99 (436)	0.21 (24)	0.10 (17)	0.98 (428)	0.35 (39)	0.18 (32)	0.92 (404)	0.12 (13)	0.06 (11)
	*PRK*	0.00 (0)	0.61 (68)	0.31 (54)	0.01 (5)	0.34 (38)	0.14 (24)	0.08 (33)	0.72 (81)	0.35 (61)
	*STA*	0.01 (1)	0.18 (20)	0.59 (103)	0.01 (4)	0.31 (35)	0.68 (118)	0.0 (0)	0.16 (18)	0.59 (102)
E3	*DR*	0.99 (281)	0.15 (17)	0.06 (5)	0.98 (277)	0.24 (27)	0.04 (3)	0.84 (238)	0.05 (6)	0.0 (0)
	*PRK*	0.01 (2)	0.73 (82)	0.03 (2)	0.01 (4)	0.24 (26)	0.03 (2)	0.15 (43)	0.65 (73)	0.53 (41)
	*STA*	0.00 (1)	0.12 (14)	0.91 (70)	0.01 (3)	0.53 (60)	0.94 (72)	0.01 (3)	0.3 (34)	0.47 (36)

**Table 8:** Sensitivity (SEN), specificity (SPE) and accuracy (ACC) of the candidates for the fine-grained classifier along with their number of true positives (TP), false positives (FP), true negatives (TN) and false negatives (FN).

	*DR*			*PRK*			*STA*		
Meas.	*RF*	*SVM*	*FRC*	*RF*	*SVM*	*FRC*	*RF*	*SVM*	*FRC*
TP	*1491*	1458	1355	215	96	*225*	356	*366*	291
TN	708	647	*748*	1873	*1909*	1685	*1803*	1706	1780
FN	*32*	65	68	142	261	*132*	106	*96*	171
FP	144	172	*71*	*92*	112	300	*77*	174	100
SEN	**0.98**	0.96	0.89	0.60	0.27	**0.63**	0.77	**0.79**	0.63
SPE	0.83	0.76	**0.88**	0.93	**0.97**	0.35	**0.96**	0.91	0.95
ACC	**0.93**	0.89	0.89	0.90	0.86	**0.92**	**0.96**	0.88	0.88

**Table 9:** Test to ensure the homoscedasticity and the normal distribution of the evaluation results for the coarse-grained classifiers.

Test	*p*-Value
Levene	0.82
Shapiro-Wilk	0.80

**Table 10:** Dunn test for coarse-grained classifiers.

		RF	FRC
FRC	Mean Diff.	−2.39	-
	*p*-value	0.03	-
SVM	Mean Diff.	−0.75	1.64
	*p*-value	0.23	0.10

**Table 11:** Test to ensure the homoscedasticity and normal distribution of the evaluation results for the fine-grained classifier.

Test	*p*-Value
Levene	0.65
Shapiro–Wilk	0.67

**Table 12:** Dunn test for the fine-grained classifiers.

		RF	FRC
FRC	Mean Diff.	−2.68	-
	*p*-value	0.01	-
SVM	Mean Diff.	−1.34	1.34
	*p*-value	0.18	0.09

**Table 13:** Summary of the evaluation results with and without breakout detection.

	With Breakout		Without Breakout
Exp.	Accuracy	Savings	Accuracy
E1	0.89	0.25	0.89
E2	0.80	0.28	0.84
E3	0.87	0.23	0.90
**Mean**	**0.85**	**0.25**	**0.88**

**Table 14:** System’s confusion matrix for the experiments in terms of percentage and number of instances (in brackets). SBDA, speed-based breakout detector agent.

		SBDA Disabled			SBDA Enabled		
Exp.	Man.	*DR*	*PRK*	*STA*	*DR*	*PRK*	*STA*
E1	*DR*	0.97 (778)	0.30 (39)	0.09 (18)	0.98 (785)	0.33 (43)	0.09 (19)
	*PRK*	0.02 (17)	0.45 (60)	0.06 (12)	0.01 (10)	0.45 (59)	0.06 (12)
	*STA*	0.01 (7)	0.25 (33)	0.86 (181)	0.01 (7)	0.23 (30)	0.85 (180)
E2	*DR*	1.00 (436)	0.25 (28)	0.13 (22)	1.00 (436)	0.43 (48)	0.23 (40)
	*PRK*	0.00 (0)	0.60 (67)	0.28 (49)	0.00 (0)	0.50 (56)	0.26 (46)
	*STA*	0.00 (1)	0.15 (17)	0.59 (103)	0.00 (1)	0.07 (8)	0.51 (88)
E3	*DR*	0.99 (281)	0.20 (23)	0.06 (5)	1.00 (283)	0.26 (29)	0.25 (19)
	*PRK*	0.01 (2)	0.67 (76)	0.03 (2)	0.00 (1)	0.63 (71)	0.03 (2)
	*STA*	0.00 (1)	0.12 (14)	0.91 (70)	0.00 (0)	0.12 (13)	0.73 (56)

**Table 15:** Coarse-grained classifier’s confusion matrices with and without GPS enrichment.

	Accelerometer + GPS		Accelerometer	
Man.	*DR*	*NoDR*	*DR*	*NoDR*
*DR*	0.99 (8885)	0.32 (494)	0.98 (8843)	0.42 (482)
*NoDR*	0.02 (135)	0.68 (1141)	0.02 (177)	0.58 (1153)
*ACC*	0.94		0.94	

**Table 16:** Fine-grained classifier’s confusion matrices with and without GPS enrichment.

	Accelerometer + GPS			Accelerometer		
Man.	*DR*	*PRK*	*STA*	*DR*	*PRK*	*STA*
*DR*	0.99 (8943)	0.38 (261)	0.12 (111)	0.99 (8925)	0.41 (281)	0.17 (162)
*PRK*	0.00 (23)	0.50 (340)	0.19 (181)	0.01 (24)	0.49 (330)	0.23 (221)
*STA*	0.01 (54)	0.12 (81)	0.69 (660)	0.01 (71)	0.10 (71)	0.60 (570)
*ACC*	0.88			0.86		

**Table 17:** System’s confusion matrices with and without GPS enrichment.

	Accelerometer + GPS			Accelerometer		
Man.	*DR*	*PRK*	*STA*	*DR*	*PRK*	*STA*
*DR*	0.98 (8966)	0.27 (180)	0.26 (251)	0.97 (8975)	0.21 (140)	0.39 (372)
*PRK*	0.01 (24)	0.70 (482)	0.16 (151)	0.02 (22)	0.75 (511)	0.12 (111)
*STA*	0.01 (31)	0.03 (20)	0.58 (551)	0.01 (23)	0.04 (31)	0.49 (470)
*ACC*	0.86			0.85		

**Table 18:** Distribution of instances among the maneuvers of the circuit with the new driver in terms of the percentage and total number (in brackets).

Maneuver	*DR*	*PRD*	*PRK*	*ST*	Total
*circuit_new-user_*	54.11 (25,159)	15.84 (7368)	26.48 (12,312)	3.57 (1655)	46,494

**Table 19:** Confusion matrices when the system is used by a driver who is different or the same as the one for which the system was trained.

	Different Driver			Same Driver		
Man.	*DR*	*PRK*	*STA*	*DR*	*PRK*	*STA*
*DR*	0.99 (977)	0.96 (498)	0.89 (333)	0.99 (1504)	0.34 (120)	0.17 (78)
*PRK*	0.00 (3)	0.03 (17)	0.09 (34)	0.01 (11)	0.52 (186)	0.13 (60)
*STA*	0.00 (2)	0.01 (3)	0.02 (4)	0.00 (8)	0.14 (51)	0.70 (324)
*ACC*	0.53			0.85		
